# Interaction-aware agent-based simulation of customer trajectories in retail stores with transformer architectures

**DOI:** 10.1038/s41598-025-22885-4

**Published:** 2025-11-06

**Authors:** Taizo Horikomi, Takayuki Mizuno

**Affiliations:** 1 The Graduate University for Advanced Studies, SOKENDAI, Shonan Village, Hayama, Kanagawa 240-0193 Japan; 2 National Institute of Informatics, 2-1-2 Hitotsubashi, Tokyo, 101-0003 Japan

**Keywords:** Consumer behavior, Agent-based simulation, Transformer model, Interaction-aware modeling, Data-driven modeling, Retail analytics, Computational science, Human behaviour, Phase transitions and critical phenomena

## Abstract

We propose a Transformer-based generative model that learns socially responsive customer trajectories in retail stores directly from data. Each trajectory is represented as a sequence of symbolic tokens that encode not only the self-location of the focal customer but also the positions of their nearest neighbors at each timestep. This interaction-aware encoding enables the model to reproduce adaptive behaviors—such as slowing down, rerouting, and early disengagement—without predefined rules. To ensure that only the focal customer’s behavior is learned while using neighbors as context, we introduce an asymmetric loss masking scheme that excludes non-focal tokens from prediction targets. The model is trained from scratch using high-resolution indoor positioning data and validated through large-scale agent-based simulations under varying crowding levels. In these simulations, each agent is equipped with a Transformer module that predicts its next step based on local spatial context, enabling the system to evolve through decentralized, data-driven decision-making. The model replicates spatial density patterns, dwell time distributions, and congestion-induced speed reductions observed in real stores. This model offers a scalable and interpretable approach to trajectory generation in indoor commercial environments.

## Introduction

In the retail industry, understanding how customers move within stores is essential for optimizing layouts, managing congestion, and enhancing the overall shopping experience^1^. In the era of digital twins^2,3^, simulation plays a critical role in reproducing in-store customer movement patterns under varying conditions. Such simulations are used to evaluate hypothetical layout changes, predict bottlenecks during peak hours, and design adaptive interventions such as dynamic signage or routing strategies in smart retail systems. These practical applications also provide a basis for connecting customer movement to broader behavioral dynamics.

From a behavioral science perspective, customer movement within stores is shaped not only by individual preferences but also by contextual constraints, including crowding and the presence of other shoppers^4^. These factors often trigger adaptive behaviors such as hesitation, detouring, or slowing down—responses that are socially sensitive and may not follow linear decision patterns. In particular, studies in pedestrian dynamics have documented phenomena such as lane formation and congestion-induced rerouting, which arise from local interactions rather than centralized planning^5,6^. Similar dynamics can be observed in retail stores, where customers instinctively adjust their paths in response to the movement and proximity of nearby individuals.

Conventional modeling approaches for human movement can be broadly categorized into macroscopic and microscopic models. Macroscopic models, such as the Continuum Theory^7^, treat pedestrian flow as a continuous fluid, capturing large-scale phenomena like density waves or bottlenecks. Microscopic models, including the Social Force Model (SFM)^8^ and cellular automata (CA)^9^, simulate individual agents based on predefined local rules or interaction forces. While these approaches have yielded useful insights, their reliance on hand-crafted rules often limits realism and scalability, particularly in complex retail environments with heterogeneous layouts and diverse customer behaviors.

To overcome these limitations, recent efforts have begun to replace rule-based agent logic with deep learning models that can learn behavior patterns directly from data. By doing so, each agent’s decision-making process becomes context-sensitive and adaptive, enabling more flexible and realistic simulations^10–12^. Alternative architectures such as Graph Neural Networks (GNNs)^13,14^ and Multi-Range Transformers^15^ have also been explored for capturing spatial interactions and long-range dependencies. While GNNs are well-suited for representing relational structures, they often face challenges in modeling long-range sequential dependencies, which are crucial for reproducing human-like trajectories. Moreover, both GNNs and hierarchical models typically require explicitly defined spatial or interaction graphs, which can limit their adaptability in dynamic or data-driven settings. In contrast, our approach adopts a standard Transformer architecture to fully leverage the powerful context modeling capabilities of large language models (LLMs). Transformer-based models, when scaled and trained on large text corpora, form the foundation of LLMs that excel in contextual reasoning. This design choice reflects a preference for architectural simplicity, interpretability, and the ability to operate without predefined structural assumptions, rather than a claim of superiority over alternative methods.

Autoregressive Transformer-based architectures have been shown to excel at long-sequence generation tasks^16,17^, making them well suited for modeling human movement patterns. Our previous work^18^ built upon this property and introduced a GPT-2-based generative model trained from scratch to reproduce in-store customer trajectories. While that model was able to generate realistic trajectories, capturing stop-and-go behaviors and route variability observed in real data, it did not reproduce adaptive responses to in-store crowding. For practical applications such as evaluating layout changes or congestion management, the ability to simulate behavioral adaptations under crowded conditions is essential, and thus the previous framework remained insufficient. Building upon this limitation, the present study extends the modeling framework to incorporate local social interactions by conditioning trajectory generation on the positions of nearby customers.

However, our previous model and similar approaches generally treat each agent as an isolated decision-maker, generating trajectories without explicitly considering the presence or movement of others. While this simplification may be sufficient in sparse or low-interaction settings, it falls short in crowded environments such as retail stores, where customers continually adapt to nearby individuals through yielding, rerouting, or slowing down.

To address this gap, we introduce a novel interaction-aware generative model that explicitly incorporates local social context into each token. Each token encodes not only the current position of the focal customer but also the positions of their two nearest neighbors at each timestep. This enriched representation allows the model to learn socially adaptive behavior patterns—such as dynamic avoidance or coordination—without relying on predefined interaction rules. In contrast to prior data-driven models, our approach directly embeds interaction sensitivity into the generative process itself, enabling scalable and realistic simulations of crowd-responsive behavior in retail environments.

To empirically validate our approach, we conducted large-scale agent-based simulations across eight crowding conditions, defined by varying customer arrival rates (i.e., number of customers entering the store per minute). The model was trained from scratch using high-resolution indoor positioning data and was not given any additional information such as store layout, product placement, or customer destinations. During simulation, each agent generated its trajectory in an autoregressive manner, conditioned only on its own past trajectory and the dynamic positions of its two nearest neighbors. The resulting movement patterns were analyzed using both microscopic indicators (e.g., turning behavior) and macroscopic metrics (e.g., dwell time, spatial density). A comparative experiment with different numbers of referenced neighbors demonstrated the critical role of social context in generating human-like movement.

In summary, the primary contribution of this study is to demonstrate that explicitly embedding local social context into a Transformer-based generative model enables realistic simulation of in-store customer trajectories. This capability provides a foundation for prospective practical applications such as digital twin systems for store layout evaluation and congestion management, while the main focus of this paper is to validate the modeling approach itself. The symbolic token representation, which encodes both spatial and social context, offers a flexible foundation for future extensions. These include explicit modeling of group-level coordination—where multiple customers move together based on shared goals or verbal interactions—as a natural extension of the implicit responses already learned by our model, such as yielding or rerouting in response to nearby individuals. Further enhancements could incorporate contextual conditioning, such as time-of-day effects or customer attributes, as well as multimodal signals like gaze direction or product interaction events (e.g., reaching for or placing an item in a basket).

The remainder of this paper is organized as follows. We begin by detailing our proposed interaction-aware generative model in the Results section, as the modeling architecture constitutes a key contribution of this work. We then present large-scale simulation results across varying customer density scenarios to demonstrate the model’s ability to reproduce realistic movement. In the Discussion, we interpret the behavioral patterns, assess the role of social context, and discuss limitations and directions for future research. Finally, the Methods section provides supplementary technical details, including the dataset, tokenization process, and simulation configurations.

## Results

### Overview of the proposed model

We first provide an overview of the proposed model to clarify how it differs from previous work. The detailed architecture, tokenization process, and training procedures are described in the Methods section.

The model generates the next position of a focal customer while conditioning on the positions of the two nearest neighbors at each timestep. This interaction-aware design enables the model to learn socially responsive behaviors, which are evaluated in the following sections. In the subsequent analyses, we evaluate the model’s ability to reproduce key behavioral patterns observed in real customer trajectories under varying levels of in-store crowding.

### Reproduction of realistic behavior

**Figure 1:**
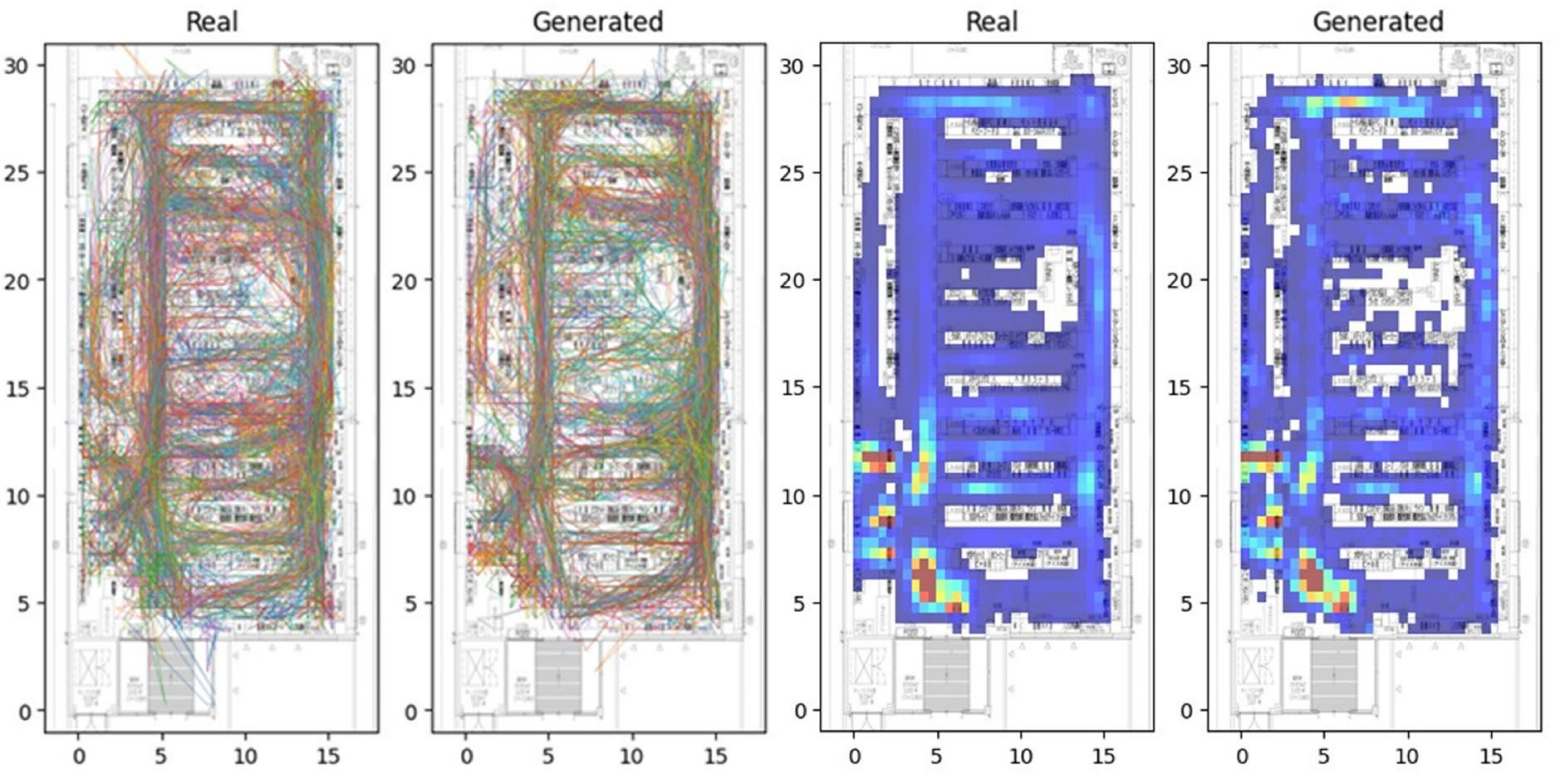
Comparison of real and generated customer trajectories and spatial densities. Left: Overlaid trajectories of 200 customers in the real dataset (leftmost) and in the simulation (second from left). Right: Heatmaps of space occupancy. High-density areas near shelves and aisles are similarly captured, indicating realistic flow patterns.

We first assessed whether the proposed model could reproduce realistic patterns of customer behavior under typical operating conditions. Figure 1 presents qualitative comparisons between the real dataset and model-generated trajectories. On the left, 200 customer trajectories are overlaid to visualize movement dynamics and corridor usage. The generated trajectories exhibit realistic circulation patterns, especially around central aisles and entrance areas, closely resembling those observed in the real environment.

The right panel shows heatmaps of spatial occupancy. The empirical heatmap was created from approximately 42,000 customer trajectories, while the simulated one was based on 1,000 generated trajectories under low to moderate crowding conditions. Despite the difference in sample size, the simulation reproduces high-density zones such as around product shelves and narrow aisles, indicating strong spatial fidelity in modeling crowd flow and location preference.

**Figure 2:**
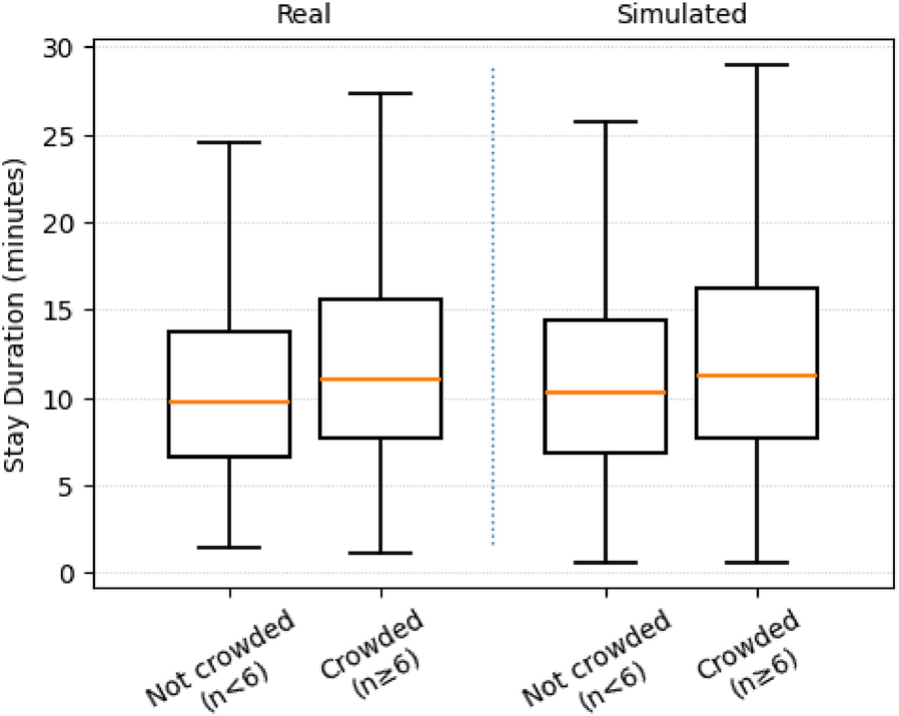
Stay duration distributions under different crowding levels. The simulation replicates increased dwell times under crowding. Boxplots show the interquartile range and whiskers without plotting outliers for visual clarity.

In addition to spatial patterns, the model captures temporal characteristics of customer behavior. Figure 2 compares the distribution of in-store stay durations between the real and simulated data using boxplots. Both datasets show similar medians and interquartile ranges, with positively skewed tails. Under low-density conditions $(n < 6)$, their central ranges largely overlap. As density increases $(n \ge 6)$, dwell times become longer in both datasets, indicating that the model learns to simulate congestion-induced slowdowns and pauses in customer movement^6,19^.

**Figure 3:**
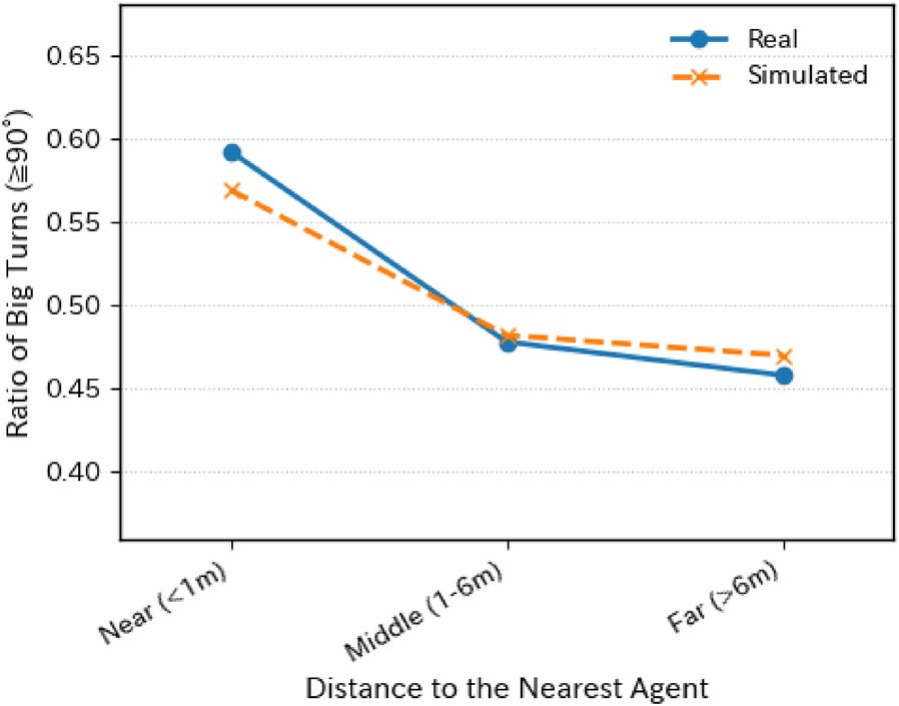
Ratio of big turns ($\ge 90^\circ$) at different distances to the nearest agent. Real (blue solid line with circles) and simulated (orange dashed line with crosses) data are plotted on the same axes to facilitate direct comparison. The simulation successfully reproduces the increase in sharp turns when another customer is nearby.

To further evaluate interaction sensitivity, we analyzed turning behavior as a function of inter-agent distance. Figure 3 shows the ratio of large-angle turns $(\ge 90^\circ )$ as a function of distance to the nearest agent. In both datasets, low proximity ($<1.0$ meter) is associated with a higher frequency of large-angle and U-turns, indicative of evasive maneuvers. Under low-crowding conditions, smaller turns dominate. The model successfully reproduces this pattern, demonstrating its ability to learn fine-grained behavioral adaptations.

**Figure 4:**
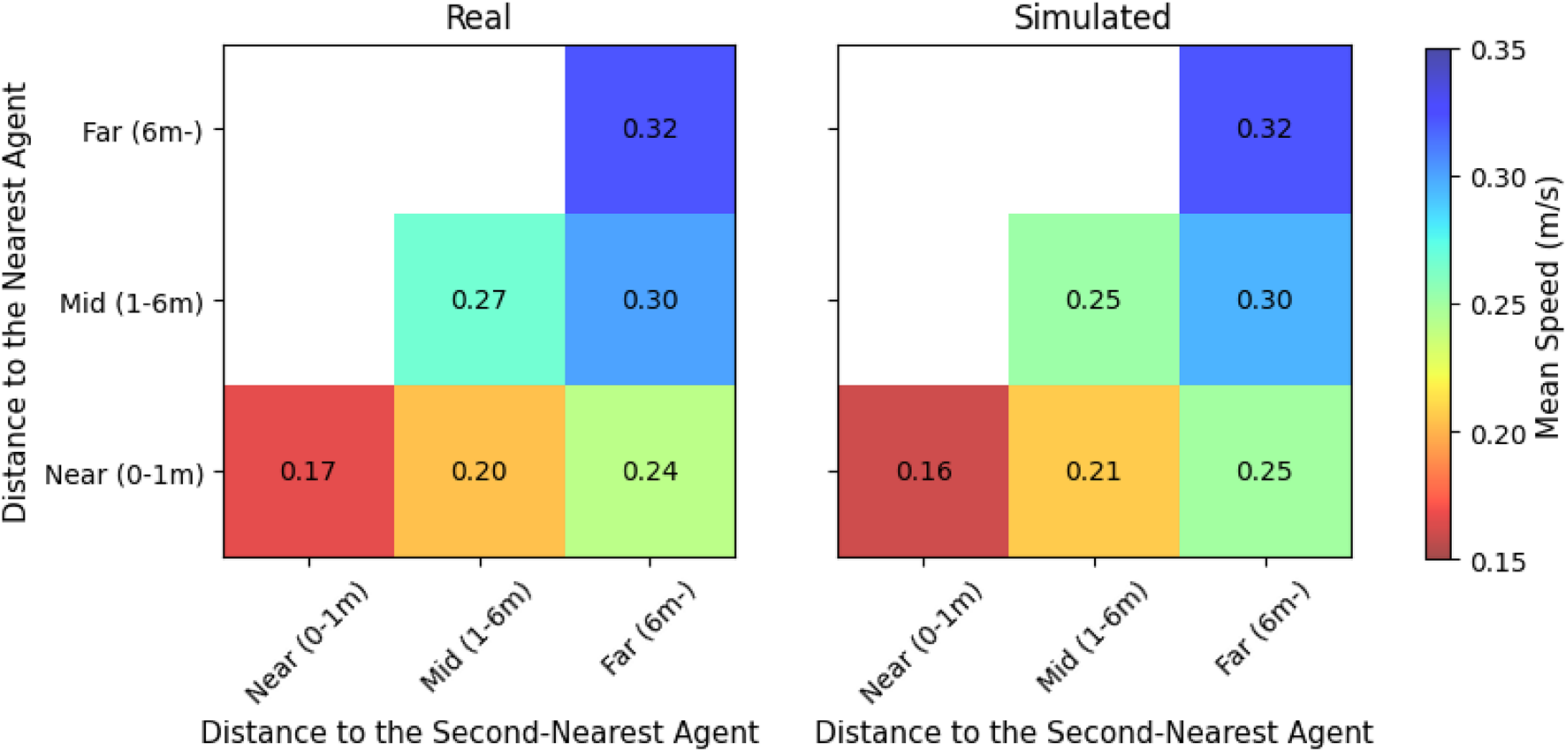
Speed modulation by spatial proximity. Left: Real data; Right: Simulated data. Each cell shows mean speed as a function of distances to nearest and second-nearest agents. Speeds decrease in high-proximity regions, indicating interaction-aware slowdowns.

Figure 4 provides additional evidence of emergent interaction effects. The heatmaps show average movement speed as a function of distances to the nearest and second-nearest agents. In both datasets, speeds decrease with increasing proximity to multiple agents, suggesting that the model effectively learns adaptive deceleration in dense local contexts.

**Figure 5:**
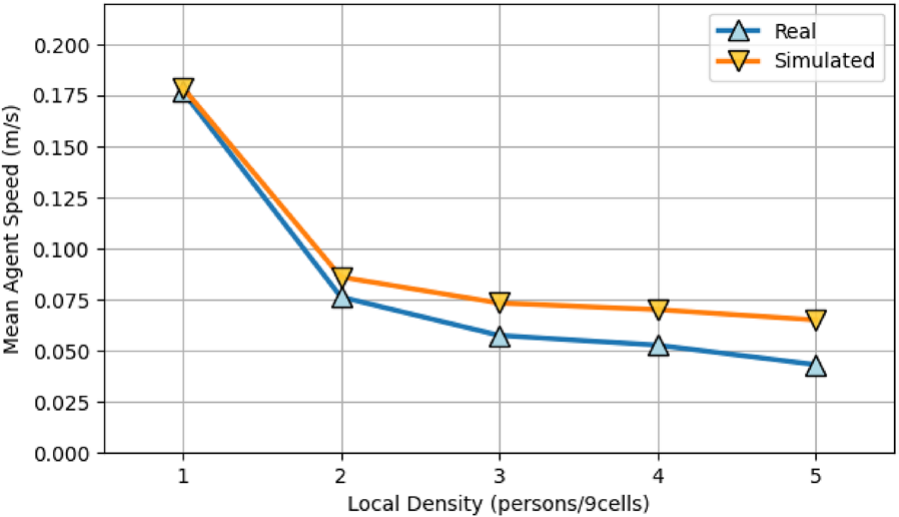
Relationship between local density and movement speed. The plot shows average movement speed as a function of local density (1–5 persons per 9-cell area) for real (upward triangle) and simulated (downward triangle) data. Both curves exhibit decreasing speed with increasing density.

Finally, we examined the relationship between local crowd density and average movement speed (Fig. 5). In both real and simulated data, speed remains stable under low-density conditions but declines nonlinearly as local density increases. The model replicates this transition pattern with high fidelity, reproducing the characteristic behavioral shift from free flow to congestion.

### Behavioral adaptations to congestion

Beyond replicating baseline behavior, our model exhibits adaptive responses to varying levels of store congestion. These behavioral adaptations emerge without any explicit programming of congestion-handling rules, indicating that the model internalizes local interactions purely from data.

To evaluate this capability, we simulated eight conditions with different customer arrival rates ranging from 0.15 to 12.0 persons per minute. While the lower four conditions approximate real-world density levels, the upper four represent stress-test scenarios beyond observed data, introduced to examine the model’s capacity to generalize and reveal nonlinear dynamics (Table 1).

**Table 1:** Simulation outcomes under varying crowding conditions.

Condition	Arrival rate (persons/min)	Avg. in-store count	Travel distance (m)	Stay duration (min)	Velocity (m/s)
Real data	–	3.26	130.5	11.49	0.189
Not crowded	0.15	1.93	129.4	10.84	0.199
Typical crowding	0.30	3.99	141.7	12.32	0.192
Busy	0.60	7.88	142.1	12.65	0.187
Very busy	1.20	15.18	137.7	12.25	0.187
Hypothetical crowding	2.40	29.18	133.1	11.79	0.188
High-density scenario	4.00	47.77	130.8	11.48	0.190
Extreme congestion	6.00	72.40	131.4	11.54	0.190
Stress-test condition	12.00	143.39	126.9	10.95	0.193

The arrival rate denotes the number of entering customers per minute. Real observations are listed at the top, followed by simulation scenarios. The upper four simulation conditions reflect plausible in-store densities observed in real settings, while the bottom four represent stress-test conditions beyond those empirically observed.

Interestingly, customer behavior did not scale linearly with increased arrival rates. Stay duration and travel distance peaked under moderate crowding but declined in highly congested settings. For instance, under the “Stress-Test Condition” with over 140 simultaneous agents, agents tended to shorten their visits and reroute to avoid congestion. This suggests a form of behavioral disengagement, in which customers adaptively terminate or redirect their paths to cope with excessive density—despite no rule-based intervention being encoded.

These nonlinear patterns resemble findings in traffic and pedestrian studies, where phase-transition-like dynamics emerge once crowding surpasses a critical threshold^19,20^. Our results suggest that such emergent responses can be captured via next-token generation alone, provided sufficient interaction-aware training data.

### Effect of the number of referenced neighbors

To further evaluate the role of social context, we trained models with different numbers of referenced neighbors ($k=0,1,2,3$). Here, $k=0$ corresponds to a non-interactive model that encodes only the focal customer’s positions, while higher values incorporate additional neighboring agents. For each *k*, we compared the simulated trajectories with the real data using the root mean squared error (RMSE) between fitted quadratic curves of stay duration or total travel distance as functions of crowding.

**Figure 6:**
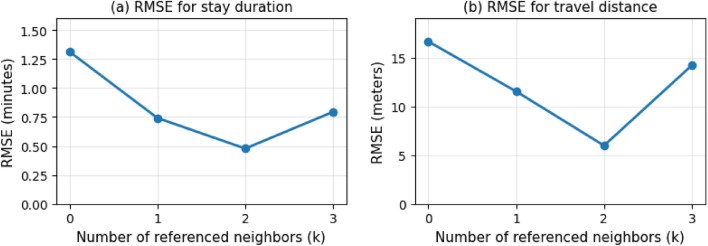
Root mean squared error (RMSE) between the polynomial fits of real and simulated trajectories, computed for different numbers of referenced neighbors (*k*). (**a**) Stay duration; (**b**) total travel distance. The error decreases from $k=0$ to $k=2$, indicating that incorporating up to two nearby agents improves behavioral fidelity, while additional neighbors provide limited or noisier benefit.

Figure 6 shows that the RMSE decreases from $k=0$ to $k=2$, reaching its minimum at $k=2$, but slightly increases again at $k=3$. These results suggest that incorporating up to two neighbors provides the best match to real data in this dataset.

### Summary of findings

The results presented above demonstrate that the proposed interaction-aware Transformer model can successfully reproduce realistic in-store customer trajectories across a wide range of crowding conditions. By encoding the positions of neighboring agents as symbolic tokens, the model learns socially adaptive behaviors—such as rerouting, deceleration, and early disengagement—without requiring any rule-based programming.

Qualitative and quantitative comparisons with real data confirm that the model captures key behavioral patterns, including spatial density distributions, dwell time variations, turning behavior, and speed modulation in response to local proximity and density. Importantly, behavioral responses change systematically with increasing arrival rates, reflecting empirically observed congestion effects. These include not only slower movement but also non-monotonic shifts in travel distance and dwell time.

Furthermore, a controlled comparison with a non-interactive variant underscores the critical importance of encoding multi-agent context. Only the interaction-aware model was able to reproduce the adaptive effects of crowding, suggesting that social interactions are a necessary condition for generating human-like movement in dense environments.

Taken together, these findings validate the effectiveness of the proposed symbolic generative approach and highlight its potential for data-driven modeling of human trajectories in complex indoor spaces.

## Discussion

This study demonstrated that a Transformer-based generative framework incorporating interaction-aware inputs can reproduce not only spatially plausible customer trajectories but also emergent behavioral adaptations under varying levels of in-store crowding. Unlike conventional rule-based approaches, our model learns directly from symbolic representations derived from fine-grained positioning data, capturing the complex interdependence between environmental constraints and social context without manual design.

A key strength of the model lies in its ability to simulate collective behavior under dynamic conditions. In the crowding simulations with varied arrival intervals, all agents were generated simultaneously using the interaction-aware model, allowing their trajectories to evolve with mutual influence in a realistic, fully generative setting.

For the ablation experiment comparing interaction-aware and non-interactive models, we simulated only a single focal agent using the model, while the surrounding agents followed their empirical trajectories. This design enabled controlled comparison under realistic density without compounding model errors.

From a behavioral science perspective, the observed nonlinear responses—such as increased dwell time and longer routes in denser settings—align with theories of bounded rationality and social friction. These results indicate that consumers do not follow fixed strategies but adapt behaviorally to the local density and motion of others. Such deceleration, avoidance, or disengagement behaviors emerged naturally from the training process, without being hard-coded, highlighting the expressive capacity of the model architecture.

This phenomenon of early disengagement can also be interpreted in light of stress-avoidance behavior under high crowding. When environmental complexity or social density exceeds a psychological threshold, customers may truncate exploration or abandon intended goals—not due to physical obstruction but as a coping mechanism to reduce cognitive and emotional strain. Empirical studies on retail crowding indicate that excessive density increases stress and confusion, reduces dwell time, and shifts behavior from exploratory to goal-oriented or avoidance-focused modes^21,22^. This aligns with the concept of “satisficing”—where decision-makers seek good-enough outcomes rather than optimal ones—originally proposed in bounded rationality theory^23^. Our findings provide empirical support that such behavioral shifts emerge naturally in learned movement models, reinforcing the model’s capacity to reflect subtle aspects of consumer psychology under crowding.

The comparison across different numbers of referenced neighbors further underlines the importance of modeling social interaction. While all models shared the same architecture and training data, their performance differed markedly depending on how many neighboring agents were encoded. RMSE between fitted curves of simulated and real trajectories decreased from $k=0$ (non-interactive) to $k=2$, but increased again at $k=3$. This suggests that in the present dataset, incorporating information from up to two neighbors provides the strongest and most reliable interaction signals, whereas the third neighbor is typically more distant and contributes weaker, noisier information. This outcome reflects the spatial characteristics of the observed store: the average number of customers present (excluding empty-store intervals) was 2.38, and situations with four or more customers simultaneously present were relatively rare. Consequently, training signals for $k=3$ were limited, which explains the observed degradation in performance. Nevertheless, in more crowded environments such as large supermarkets or public venues, larger values of *k* (e.g., 3 or 4) could provide additional gains, and our framework is readily extensible to such settings. From a practical perspective, smaller *k* values also reduce computational cost, so $k=2$ offers a favorable balance between fidelity and efficiency in this case. Together, these findings reinforce that spatial context alone is insufficient for simulating realistic crowd-aware behavior, and that carefully encoding local interactions is essential for scalable, socially responsive trajectory generation.

An important limitation of this study is that the positioning data captured only customers who carried shopping baskets, due to sensor placement constraints. According to the data provider, approximately half of the store visitors do not use baskets. Prior research suggests that basket-carrying customers tend to engage in more planned or exploratory purchasing behaviors^24^. Consequently, our model may overrepresent goal-oriented shopping patterns, while underrepresenting spontaneous or minimal browsing behaviors typical of basket-free customers. This sampling bias limits the generalizability of the findings across broader customer types. To address this limitation, future work could explore the use of alternative sensing technologies such as depth cameras or LiDAR to detect customers who do not carry identifiable tracking devices. Additionally, statistical modeling techniques could be developed to infer the presence and influence of unobserved customers, enabling more comprehensive simulations in partially observable environments.

In practical terms, our framework enables scalable, data-driven simulations of human movement in retail spaces. It supports a range of applications including layout optimization, operational forecasting, and agent-based digital twin environments. The symbolic tokenization scheme further allows flexible extension to richer representations, such as agent identity, goal orientation, or interaction history, paving the way for personalized or multi-modal modeling.

Although this work centers on a retail use case, the proposed modeling paradigm generalizes to other domains involving human spatial interaction. The symbolic Transformer approach bridges natural language modeling with movement simulation, making it suitable for a range of environments including transit stations, museums, hospitals, and evacuation planning. As sensor technologies advance and behavioral datasets become increasingly accessible, such generative models will play a central role in understanding, forecasting, and designing for complex human dynamics.

## Methods

### Proposed interaction-aware generative model

We propose a Transformer-based generative modeling framework for simulating realistic customer behavior in indoor retail environments. Unlike rule-based models that rely on handcrafted heuristics, our approach learns behavioral patterns directly from spatiotemporal data in an autoregressive manner. The model is trained to generate the next token in a sequence conditioned on past movements and the positions of nearby agents.

**Symbolic** **spatial** **representation.** The store layout was discretized into a $64 \times 64$ grid, resulting in 4,096 spatial cells. Each cell was assigned a 6-character token derived from a hierarchical spatial encoding scheme^18^, where each character encodes a recursive quadrant subdivision. For example, the token bgjoqw represents a specific location with fine-grained spatial locality.

**Multi-agent** **input** **structure.** To capture social interactions, we constructed symbolic sequences containing the positions of three agents at each timestep:

- S (Self): the focal customer
- O (Other): the nearest neighbor
- T (Second Other): the second-nearest neighbor

**Figure 7:**
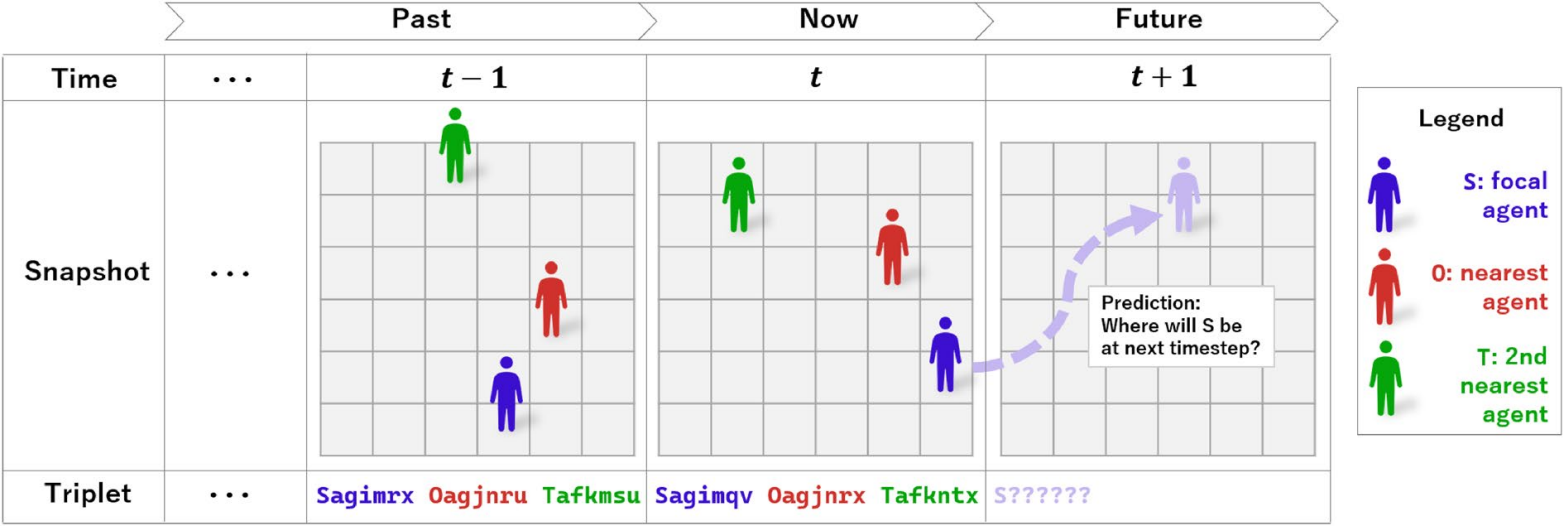
Illustration of the proposed modeling framework. At each timestep, the positions of the focal customer (S, blue), the nearest neighbor (O, red), and the second-nearest neighbor (T, green) are encoded as tokens (triplets). Each token consists of a role prefix (S=Self, O=Other, T=Second other) and a 6-character location code. The past and present sequence of triplets forms the model input, and the model predicts the next position of the focal customer at $t{+}1$ (purple dashed arrow).

Each timestep is represented as a sequence of three tokens, each composed of a role prefix and a location code. For example, if at a given timestep the focal customer is at location agimrx and their two nearest neighbors are at agjnru and afkmsu, they are represented as the tokens: Sagimrx, Oagjnru, and Tafkmsu (see Fig. 7 for an illustration).

Each token (e.g., Sagimrx) consists of a role prefix and a 6-character location. These triplets are concatenated over time to form a multi-agent sequence:

Sagimrx Oagjnru Tafkmsu Sagimrx Oagjnrx Tafkntx ...

**Asymmetric** **loss** **masking** **for socially** **grounded learning.** A key innovation of our modeling approach lies in the asymmetric loss computation that reflects the structure of social interactions. While each training input includes the positions of the focal customer (S) and two nearby individuals (O, T), only the S tokens are used as prediction targets. The O and T tokens serve exclusively as contextual signals and are explicitly masked out from the loss calculation.

This design choice captures a crucial asymmetry in real-world decision-making: agents observe others and adapt to them, but only their own behaviors are subject to intentional control and learning. By isolating the focal agent’s outputs as the sole learning targets, the model avoids spurious gradients from neighboring agents and ensures that the learned behavior reflects subjective, socially situated responses rather than averaging or imitation. This masking strategy is not merely a technical adjustment—it constitutes the foundation of socially grounded generative learning in our framework.

**Tokenization and vocabulary.** To encode the symbolic spatial sequences, we trained a Byte Pair Encoding (BPE) tokenizer^25^ from scratch on the corpus. A deliberately small vocabulary size of 200 was selected to encourage decomposition of each spatial token into 2–4 sub-tokens. This promotes compositional learning, enabling the model to generalize to unseen locations by capturing reusable substructures rather than memorizing entire tokens.

**Model architecture and training.** We adopted a GPT-2 style architecture^26^ consisting of 12 decoder layers, 12 attention heads, and a hidden size of 768. Positional embeddings were added to capture temporal structure. The model was trained from scratch using AdamW^27^ with a learning rate of $3 \times 10^{-4}$ and a batch size of 32. Learning rate scheduling and early stopping were applied based on validation loss.

To accommodate long trajectories, we extended the input length to 6144 tokens. Each training instance consisted of a continuous multi-agent sequence containing symbolic tokens for the focal agent and nearby individuals. As described above, only S tokens were used as prediction targets, and the loss was computed accordingly.

To enable efficient training on such long sequences, we employed mixed-precision training and gradient checkpointing, significantly reducing memory consumption without compromising model capacity.

### Data source

We used indoor positioning records obtained from a Japanese drugstore chain. The dataset contains timestamped sequences of customer locations captured every 5 seconds via a Quuppa-based indoor location tracking system deployed in the store^28^. Each record includes time, x/y coordinates, and an anonymous customer identifier. A total of 42,000 customer trajectories were collected over a 1-year period. Every shopping basket in the store was equipped with a dedicated tracking transmitter, ensuring that customers carrying a basket were fully captured without any missing trajectories. Due to sensor placement constraints, customers without baskets were not recorded.

### Simulation setup

To assess the performance of the proposed model, we conducted large-scale agent-based simulations that emulate various crowding conditions in a realistic indoor retail setting. Specifically, we manipulated the arrival rate of customers to produce eight distinct density levels: 9, 18, 36, 72, 144, 240, 360, and 720 customers per hour. These arrival rates reflect a range of scenarios from sparse weekday mornings to extreme hypothetical congestion.

In each simulation run, all customers were initialized with their first three observed positions from the empirical dataset. Thereafter, their movements were generated autoregressively using the trained model. At each timestep, the symbolic locations of the nearest and second-nearest neighbors were dynamically calculated and included in the input sequence as context. Generation continued until an end-of-sequence token was produced or a predefined maximum length was reached.

The overall simulation procedure is summarized in Algorithm 1. To avoid initialization artifacts, we excluded the warm-up period and focused all analyses on the equilibrium phase of the simulation, defined as the time window after the number of active customers in the store had stabilized.

**Algorithm 1:**
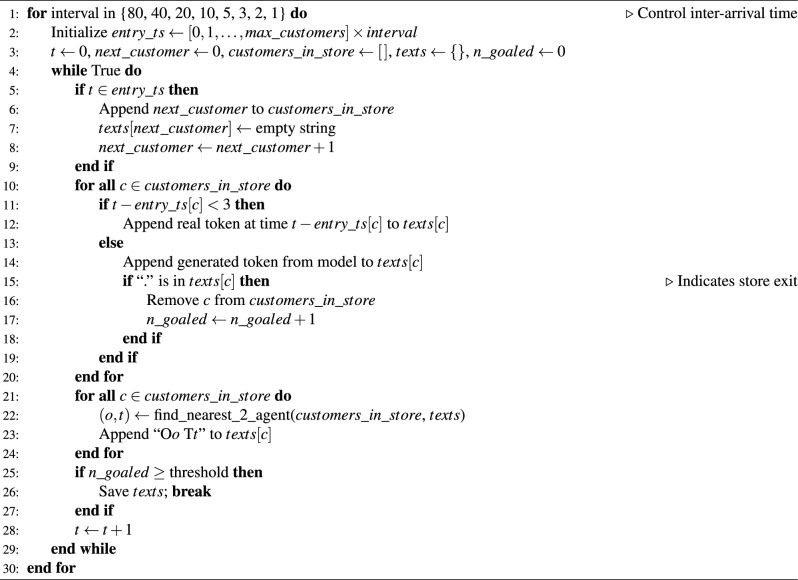
Customer simulation under varying arrival intervals

### Evaluation metrics

We evaluated the simulated trajectories using six behavioral metrics that capture key aspects of in-store customer movement. These metrics were chosen to reflect not only the realism of individual trajectories but also their relevance to practical retail applications such as store layout evaluation, congestion management, and digital twin-based operational planning.

- **Spatial density distribution**: heatmaps of cell-wise customer occupancy to assess how well the model replicates congestion-prone zones. This metric is directly relevant for store layout evaluation and congestion management in retail spaces.
- **Stay duration**: distribution of total in-store time per customer, indicating behavioral variability under different density conditions. It informs customer experience analysis and operational forecasting by capturing how long customers are likely to remain under varying crowding levels.
- **Travel distance**: cumulative spatial distance traveled, used as a proxy for route complexity. It reflects the degree of in-store exploration, which is important for evaluating store circulation design and product exposure strategies.
- **Turning angle**: distribution of directional changes between timesteps to capture motion smoothness and navigation behavior. This metric helps assess navigation safety and the efficiency of aisle layouts, which are critical for designing smooth customer flows.
- **Speed vs. local density**: average customer speed computed at varying levels of local occupancy (number of agents within a $3 \times 3$ grid), used to detect emergent slowdowns or congestion responses. It is essential for digital twin systems that simulate and prediynamic flow patterns for operational planning.
- **RMSE between fitted curves**: the root mean squared error between the polynomial approximation of the *crowding–stay duration* or *crowding–travel distance* relationship in real data and the corresponding approximation from each simulation model. This metric quantifies how closely the simulated trajectories reproduce crowding-dependent behavioral adaptations, by directly comparing fitted curves rather than raw distributions.

We compared these metrics between real and simulated trajectories to assess how well the model reproduces key behavioral patterns observed in actual in-store customer movements.

## Data Availability

The dataset used in this study consists of commercial indoor positioning records obtained from a Japanese drugstore. Due to contractual confidentiality and privacy restrictions, the full dataset cannot be made publicly available. However, the modeling pipeline, tokenization scheme, and evaluation protocol are fully described in the Methods section to enable reproduction of the modeling procedure. Aggregated or anonymized samples may be made available from the corresponding author upon reasonable request.
